# Calibrated non-inferiority margin: a new pragmatic method to account for population shift in stroke trials

**DOI:** 10.1093/esj/aakaf022

**Published:** 2026-01-01

**Authors:** Nuala Peter, Hannah Johns, Bruce C V Campbell, Bijoy Menon, Mark W Parsons, Leonid Churilov

**Affiliations:** Global Biostatistics and Data Sciences, Boehringer Ingelheim Pharma GmbH and Co. KG, Biberach an der Riss, Germany; Melbourne Medical School, University of Melbourne, Melbourne, Victoria, Australia; Australian Stroke Alliance, Melbourne Brain Centre, Royal Melbourne Hospital, Parkville, Victoria, Australia; Department of Medicine and Neurology, Melbourne Brain Centre, Royal Melbourne Hospital, University of Melbourne, Parkville, Victoria, Australia; Calgary Stroke Program, University of Calgary Cumming School of Medicine, Calgary, Alberta, Canada; Department of Neurology, Liverpool Hospital, Liverpool, New South Wales, Australia; Melbourne Medical School, University of Melbourne, Melbourne, Victoria, Australia; Australian Stroke Alliance, Melbourne Brain Centre, Royal Melbourne Hospital, Parkville, Victoria, Australia

**Keywords:** adaptive trials, non-inferiority, statistics, stroke, population shift, STTC, TASTE

## Abstract

**Introduction:**

Non-inferiority trials in acute ischemic stroke (AIS) are crucial to improve access to high-quality care. Population shifts must be accounted for when estimating non-inferiority margins, eg, changes in population characteristics (trial vs historical data); however, existing methods have practical and statistical limitations. We propose a pragmatic conceptual approach and fully pre-specifiable procedure for calibrating non-inferiority margins that account for population shifts in observed trial populations.

**Patients and methods:**

Our approach splits trial and historical data into subgroups based on relevant effect-modifying covariates. Trial data from TASTE, which investigated the effect (mRS score 0–1 at day 90) of tenecteplase vs alteplase, were compared to historical data from the Stroke Thrombolysis Trialists’ Collaboration (STTC) meta-analysis (alteplase vs control). We reweighted the STTC treatment effect to match the shifted AIS population in TASTE before deriving the calibrated non-inferiority margin.

**Results:**

For both datasets, subgroups were based on onset-to-treatment time and baseline NIHSS values. The reweighted risk difference for alteplase vs control was 11.70% (95% CI, 6.67–16.73); the conservative treatment-effect estimate was 6.67%, corresponding to a risk difference of 3.33% (50% reduction). Hence, the calibrated margin for comparing alternative interventions to alteplase was set at −3.33%, consistent with the European Stroke Organisation’s clinically recommended margin (−3.0%).

**Conclusion:**

Our conceptual approach to estimate calibrated non-inferiority margins is a simple and pragmatic alternative to existing methods to account for population shifts in stroke trials. The supporting procedure has already been applied.

## Graphical Abstract

**Graphical abstract:**
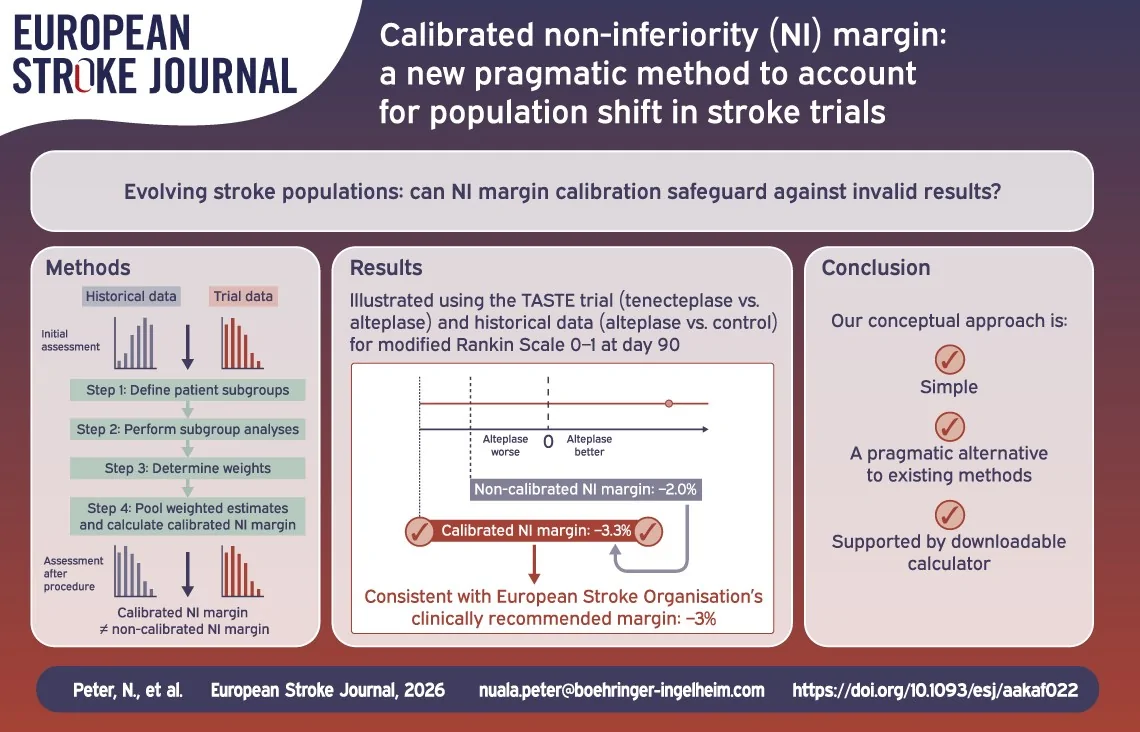

## Introduction

Non-inferiority trials in acute ischemic stroke (AIS) are vital for improving access to high-quality care through identifying alternative treatments with easier administration, reduced cost or other clinical considerations that do not unacceptably reduce treatment effect.^1^^,^^2^ Unlike standard RCTs, which are usually designed to compare an experimental treatment to a control condition (eg, standard of care or placebo), non-inferiority trials compare an experimental treatment to an active comparator. Non-inferiority margins, thresholds above which non-inferiority trials claim success, are often estimated by expert consensus^3^ or using historical data and the 95–95 fixed margin approach^4^; however, they must account for changes in patient subpopulations.^5^ Further details regarding non-inferiority margins are provided in Appendix S1 and in previous publications.^6^^,^^7^

The European Commission for Health Technology Assessments^8^ has identified several approaches to estimate the effects of experimental treatment vs control via an active comparator that account for shifts in patient populations. Although not explicitly addressed by the European Commission, in the context of non-inferiority trials such approaches inevitably rely on the derivation of calibrated non-inferiority margins that account for population changes from historical data.^8^ Each approach requires individual patient data from historical RCTs to derive these margins, or makes strong statistical assumptions that, if unsatisfied, can cause biased outcomes. In practice, legal and administrative barriers often limit data sharing,^9^^,^^10^ and statistical assumptions made from these methods are often difficult or impossible to verify.^8^

Such practical and statistical limitations hinder the use of existing methods for generating calibrated non-inferiority margins in stroke research. Given well-documented changes in effect-modifying covariates, eg, onset-to-treatment time,^11–14^ and a lack of practical approaches to handling these changes, stroke trialists face substantial barriers in specifying and justifying non-inferiority margins, despite repeated recommendations in stroke literature.^1^^,^^2^

This article aims to (1) propose a conceptual approach to calculate calibrated non-inferiority margins that account for changes in processes of care and patient demographics without relying on burdensome statistical or practical requirements; (2) describe a fully pre-specifiable procedure to support the proposed approach within non-inferiority trials and (3) illustrate this procedure using TASTE,^15^ a non-inferiority trial that compared tenecteplase to alteplase in a patient population that has meaningfully changed from historical trials that compared alteplase to control.

## Patients and methods

### Conceptual approach

**Figure 1:**
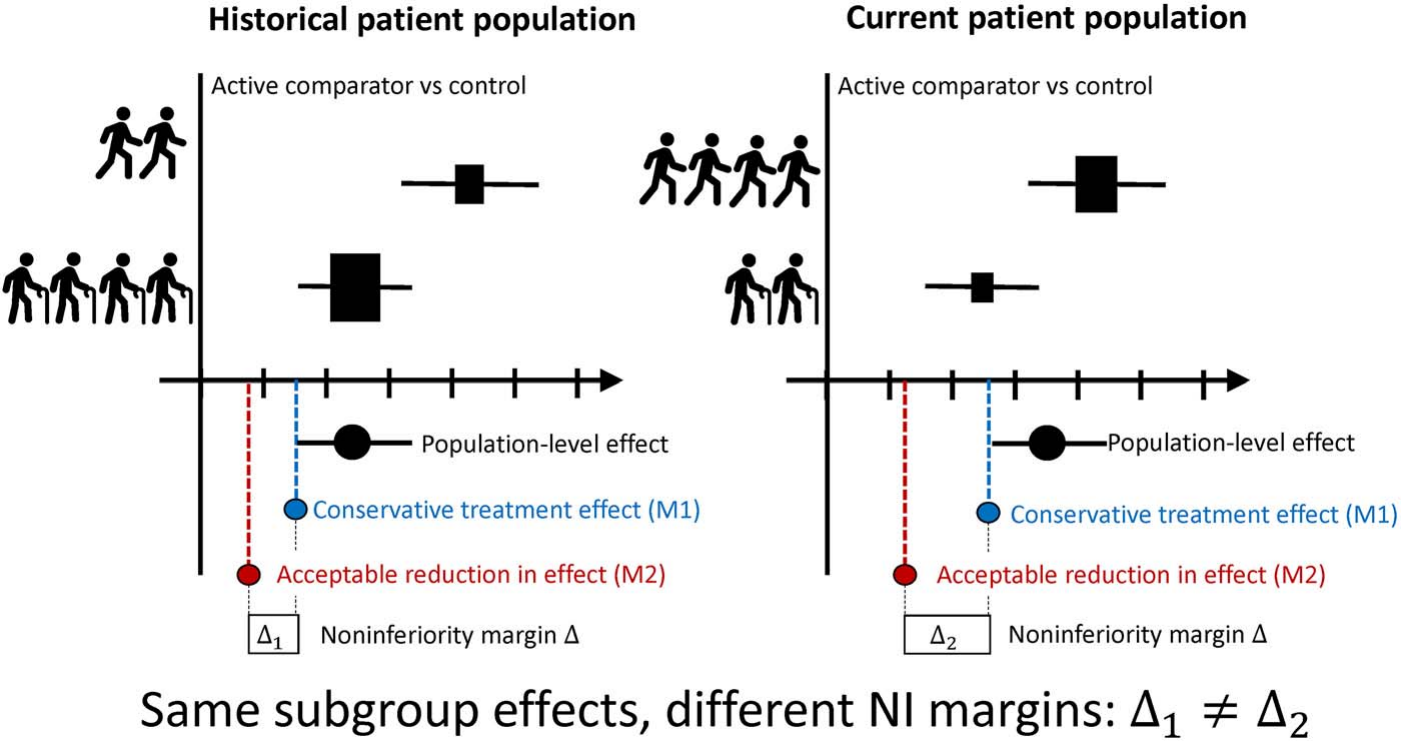
Illustration of how changes in populations impact non-inferiority margins, even if subgroup-level treatment effects remain the same. M1 and M2 terminology follows U.S. Food and Drug Administration guidance documents.^4^^,^^5^ Abbreviations: M1 = conservative estimate of treatment effect; M2 = acceptable reduction in treatment effect; NI = non-inferiority.

The fundamental challenge in non-inferiority trials outlined above is that, even if within-subgroup treatment effects remain constant over time, a potential shift in the relative frequency of these subgroups compared with the historical patient population may lead to changes in the overall treatment effect at the population level (Figure 1).

A simple conceptual approach to address this challenge is to split data into homogeneous subgroups from which subgroup-specific treatment-effect estimates can be obtained. Shifts in subgroup frequencies may be addressed by reweighting to obtain a reweighted, calibrated, pooled treatment-effect estimate that appropriately reflects population changes (M1). A calibrated non-inferiority margin that quantifies an acceptable reduction in efficacy (M2) may then be derived from this reweighted, pooled analysis using simple mathematics.

M1 reflects the full benefit of the active comparator treatment compared to control in the historical studies. M2 reflects the fraction of this benefit to be preserved in order to consider the new experimental treatment as being non-inferior to active comparator. For example, if the fraction to be preserved is set to 100%, this is the equivalent of testing if the new treatment is superior to the active comparator. If the fraction preserved is set to 0%, this is the equivalent of testing if the new treatment is superior to the control. Commonly, 50% preservation is used, but that is somewhat arbitrary. The more benefit to be preserved, the smaller the margin, and the harder for the new experimental treatment to meet the non-inferiority threshold.

Subgroup-specific treatment-effect estimates may be estimated from historical data in collaboration with data holders, published subgroup analyses or expert consensus, depending on data access.

### Design of a practical, fully pre-specifiable procedure for deriving calibrated non-inferiority margins

**Figure 2:**
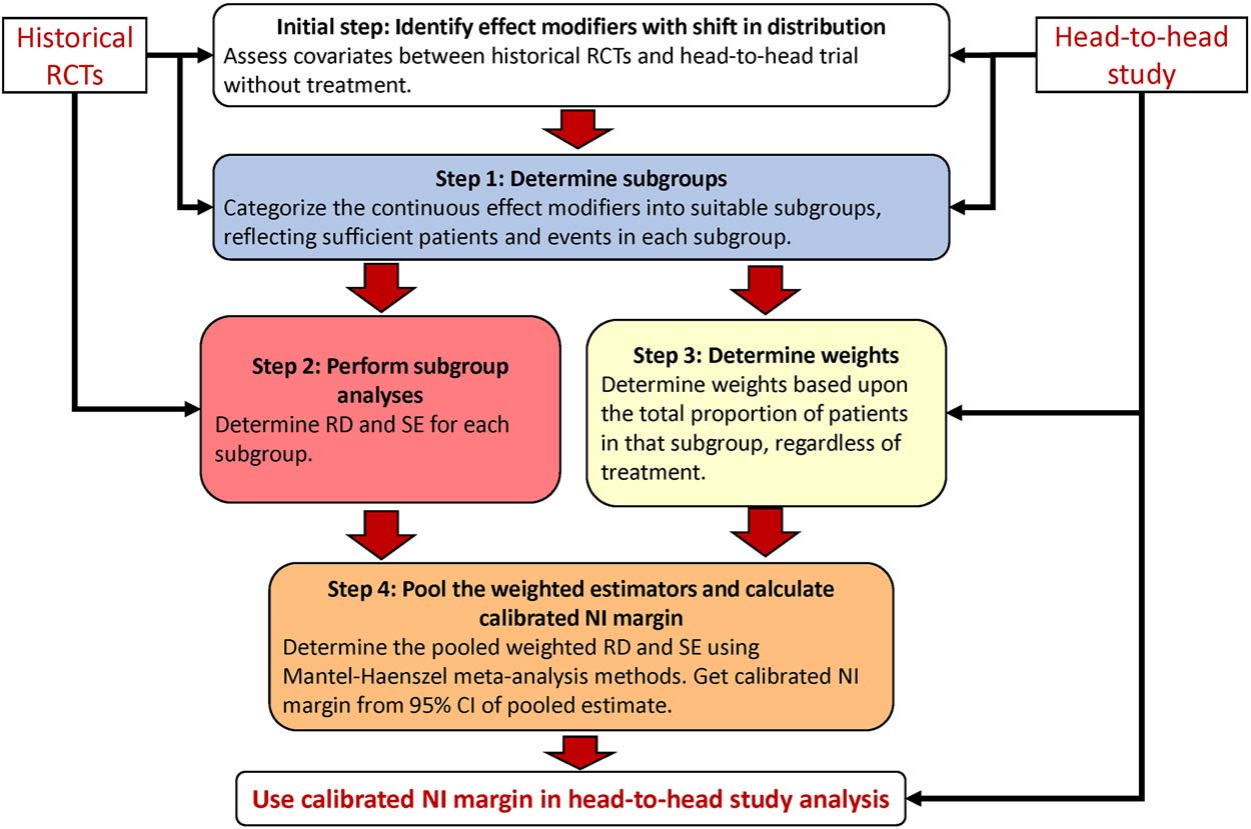
Overview of proposed procedure for estimating non-inferiority margins. Abbreviations: NI = non-inferiority; RD = risk difference; SE = standard error.

Figure 2 presents a fully pre-specifiable procedure for estimating calibrated non-inferiority margins, consisting of 4 easily performed steps. Initially, effect modifiers that result in a shift in patient populations must be identified by comparing the distribution of covariates in the historical dataset with the trial dataset. In step 1, patient subgroups are created based on the combinations of effect-modifying covariates of interest. Subgroups must be large enough to estimate subgroup-specific treatment effects within the historical dataset. A common guiding principle is that each arm within each subgroup should contain ≥ 15 observations and ≥ 1 event.

Step 2 involves estimating active comparator treatment effects within each subgroup. In step 3, weights are determined based on the relative proportion of patients in each subgroup, irrespective of treatment arm. Step 4 involves pooling the weighted meta-analysis estimators to produce a calibrated control to active comparator treatment-effect estimate in a target study population. A calibrated non-inferiority margin can then be calculated from this reweighted treatment-effect estimate using, for example, the classical 95–50–95 fixed margin rule^4^ (Appendix S1 and Table S1) or expert consensus.^3^ A complete description of the procedure, including a summary of statistical assumptions, is provided in Method S1.

### Illustrative example using TASTE

We illustrate our proposed approach by deriving a calibrated non-inferiority margin for the TASTE trial^15^ using data from the Stroke Thrombolysis Trialists’ Collaborative (STTC) meta-analysis.^16^ TASTE was a non-inferiority trial that estimated the effect of tenecteplase vs alteplase in patients, selected by use of perfusion imaging within 4.5 hours of onset of AIS, between March 2014 and October 2023, using an absolute non-inferiority margin of −3%. The design of TASTE has been published previously.^15^ The STTC dataset included patients with AIS from 9 RCTs that compared the effect of alteplase (an active comparator in the TASTE trial) vs control^16^; these were the only completed phase III RCTs of intravenous alteplase in AIS for which data were available at the time of analysis (2014). To derive the calibrated non-inferiority margin for TASTE, we reweighted the treatment effect from this meta-analysis to the population observed in the TASTE trial. The details of how each step of our procedure was applied are reported in Method S2. Although the risk difference in the intention-to-treat analysis of the TASTE trial was adjusted for age, baseline NIHSS score and pre-morbid mRS score, subgroup effects in our analysis were unadjusted but calibrated.

To assess the robustness of our 4-step approach, we conducted systematic and comprehensive sensitivity analyses. The propensity score stratification (PSS) method^17–19^ was used to calibrate the covariates using individual patient covariate data. For completeness, we employed propensity score weighting^17–19^ with and without trimming, as well as additional subgrouping of the proposed 4-step method to contrast with the results of the PSS method.

## Results

The STTC and TASTE datasets included 4361 and 680 patients, respectively. There was a shift in onset-to-treatment time between STTC and TASTE, with substantially more participants in TASTE receiving treatment within 3 hours of stroke onset (Table 1). This shift was anticipated, reflecting well-documented changes in clinical practice.^11–14^ TASTE participants also had milder baseline NIHSS scores; however, the age distribution was similar. We therefore selected onset-to-treatment time and baseline NIHSS as treatment-modifying covariates to form subgroups.

**Table 1:** Distribution of important prognostic factors in meta-analysis and TASTE

	**STTC (*n* = 4 361)**	**TASTE (*n* = 680)**
**Onset-to-treatment time**		
Median [IQR], hours	213 [165–242]	153 [124–197]
0–1.5	330 (8%)	45 (7%)
>1.5–2.0	151 (3%)	114 (17%)
>2.0–2.5	390 (9%)	166 (25%)
>2.5–3.0	678 (16%)	133 (20%)
>3.0–3.75	1 042 (24%)	114 (17%)
>3.75–4.5	1 770 (41%)	103 (15%)
**Age**		
Median [IQR], years	73 [64–82]	74 [64–81]
≤80	3 089 (71%)	493 (72%)
>80	1 272 (29%)	187 (28%)
**Baseline NIHSS**		
Median [IQR]	12 [7–17]	7 [4–11]
0–8	1 452 (33%)	429 (63%)
9–15	1 461 (34%)	162 (24%)
≥16	1 448 (33%)	89 (13%)

STTC summaries calculated from subset of data within 0–4.5 hours.

Abbreviation: STTC = Stroke Thrombolysis Trialists’ Collaboration.

### Step 1: Creating subgroups

**Figure 3:**
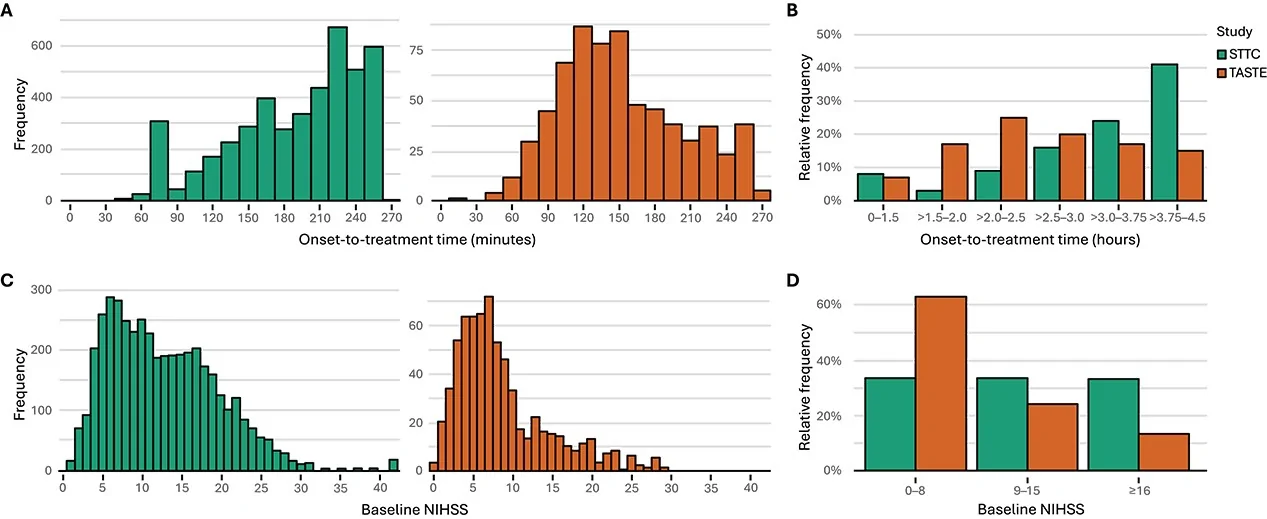
Distribution of prognostic factors in the historical meta-analysis and the new trial. Panels A and B pertain to both STTC and TASTE datasets, showing distribution across OTT categories. Panels C and D pertain to both STTC and TASTE datasets, showing distribution across baseline NIHSS categories. Abbreviation: STTC = Stroke Thrombolysis Trialists’ Collaboration.

Following the procedure described in “Patients and methods,” we examined onset-to-treatment in 15-minute time bins and baseline NIHSS in single-unit bins (Figure 3A and C), then combined these bins into clinically meaningful categories that were large enough to estimate subgroup-specific treatment effects within the STTC dataset (Figure 3B and D). This resulted in 6 onset-to-treatment time (0–1.5 hours; > 1.5–2.0 hours; > 2.0–2.5 hours; > 2.5–3.0 hours; > 3.0–3.75 hours; > 3.75–4.5 hours) and 3 baseline NIHSS (0–8; 9–15; ≥ 16) categories. More bins were used for onset-to-treatment time as treatment-effect heterogeneity was greater in these subgroups.^16^ The full-factorial combination of these bins resulted in 18 subgroups. Compared to STTC, TASTE had more participants with mild NIHSS and onset-to-treatment times of 1.5–2.5 hours.

### Step 2: Subgroup analysis results

**Figure 4:**
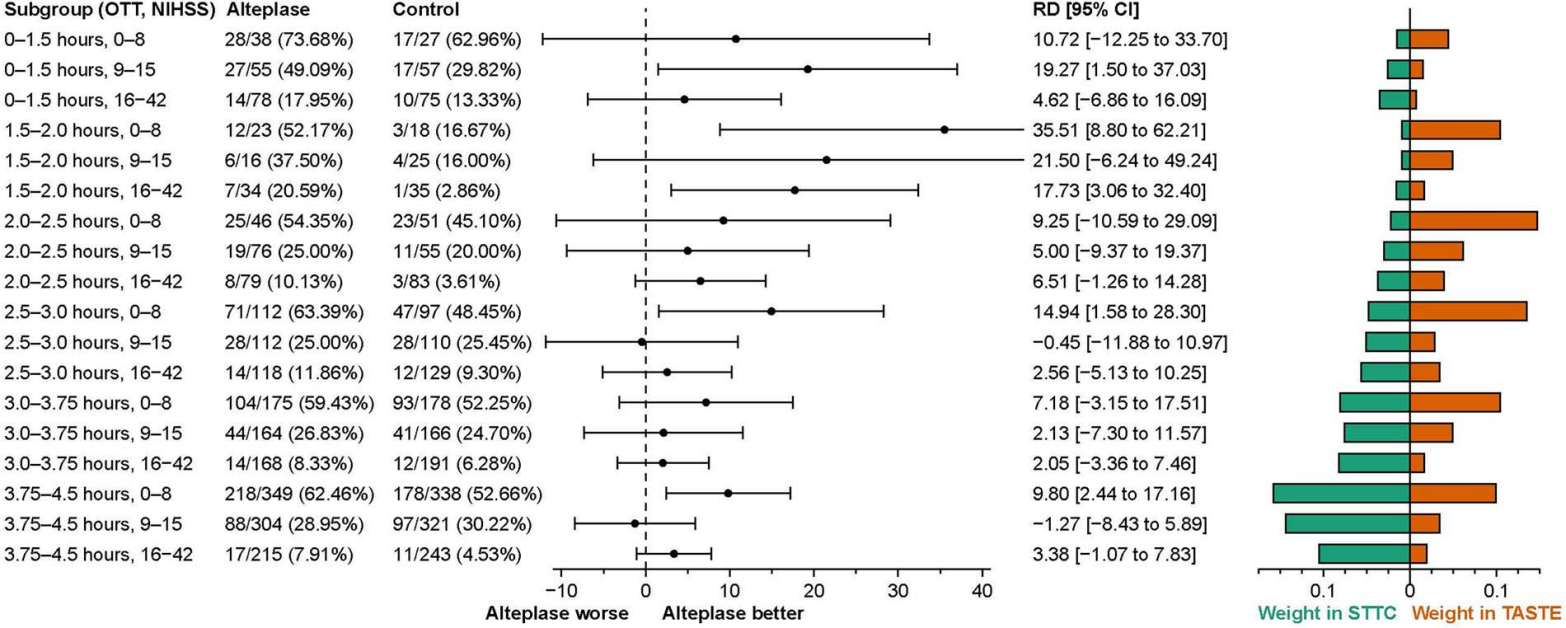
RDs and 95% CIs for mRS score of 0–1 at day 90 in the STTC data, with weights in STTC and TASTE. The figure presents re-analysis of the primary endpoint (mRS score of 0–1 at day 90) in the historical patient population (STTC database) within each of the 18 subgroups derived from Figure 3. RDs and corresponding 95% CIs were estimated for each created subgroup. Abbreviations: h = hours; OTT = onset-to-treatment time; RD = risk difference; STTC = Stroke Thrombolysis Trialists’ Collaboration.

The primary endpoint of mRS score of 0–1 at day 90 in the historical meta-analysis (STTC database) was re-analysed within each of the 18 subgroups, with the risk difference and corresponding standard error estimated for each created subgroup (Figure 4).

The estimated effect of alteplase when compared with control varied significantly across each subgroup, with higher efficacy in patients presenting with an NIHSS score of 0–8 with onset-to-treatment time of 1.5–2 or 2–2.5 hours, and no significant effect for patients presenting with an NIHSS score of 9–15 or ≥ 16 with onset-to-treatment time of 3.75–4.5 hours. Subgroups with high efficacy tended to have a higher proportion of participants within the TASTE vs STTC dataset; similarly, subgroups with low efficacy tended to have a lower proportion of participants in the TASTE vs STTC dataset. The assumption of constancy (ie, that the active comparator to control treatment effect is unchanged across study populations) was therefore violated. If the historical STTC dataset contained the same distribution of patients as those that were recruited into TASTE, we would have observed a larger treatment effect of alteplase compared with control, and we would have subsequently obtained a larger absolute magnitude for the non-inferiority margin. This cannot be ignored, as using an uncalibrated non-inferiority margin would bias the results of the non-inferiority analysis.

### Step 3: Determining weights

For each study and within each subgroup (defined in step 1), we determined the weights by calculating the proportion of data within the whole study that belonged to the subgroup, irrespective of treatment assignment. Only the weights from TASTE were required to estimate the calibrated non-inferiority margin. The weights from STTC were used together with the weights from TASTE to illustrate the notion of population shift. Differences in these weights reflect the change in population between the TASTE and STTC datasets, with substantially higher weights placed on subgroups with mild NIHSS and fast onset-to-treatment times, and reduced weight placed on subgroups with severe NIHSS (Table S2).

### Step 4: Pooled effect estimate and calibrated non-inferiority margin

**Figure 5:**
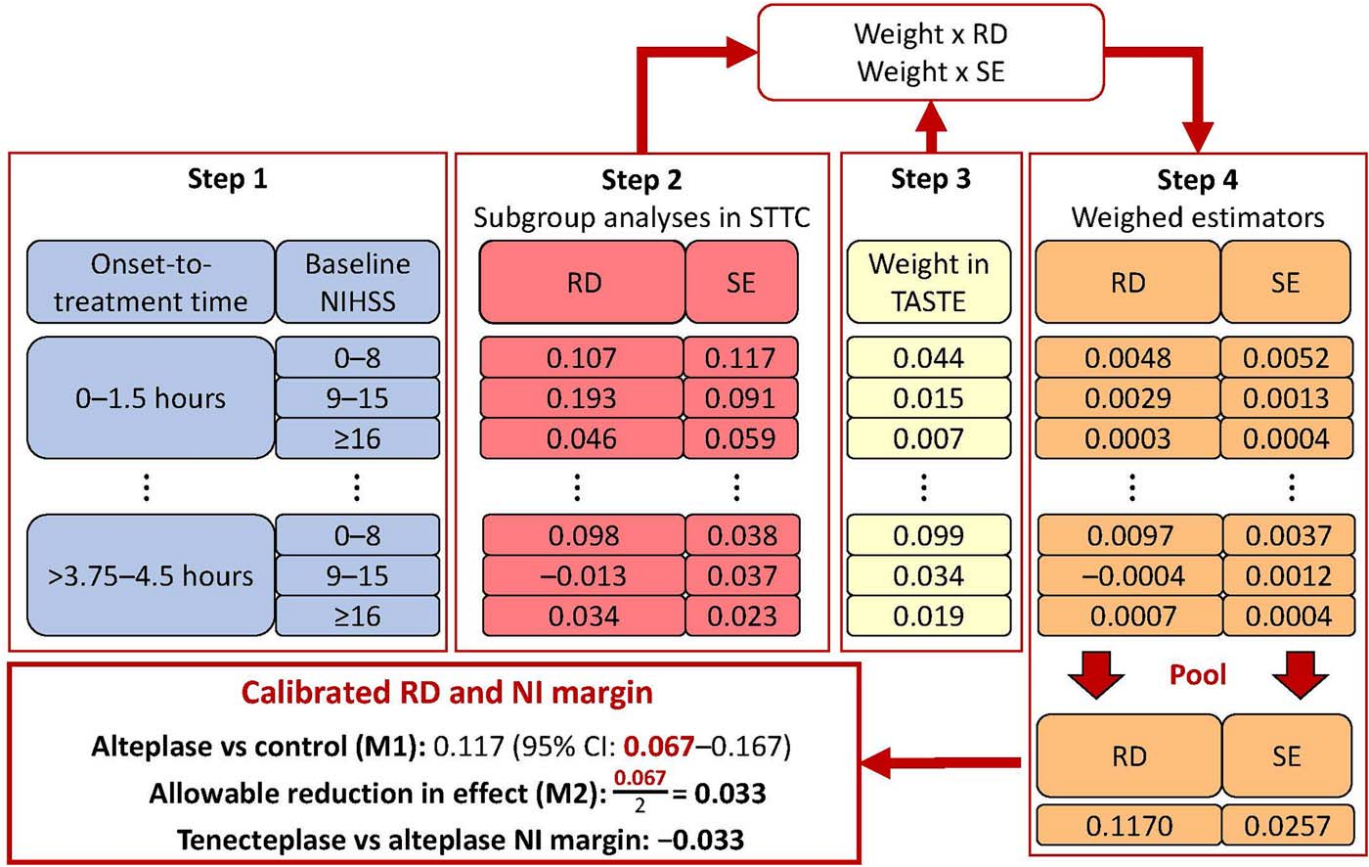
Summary of derivation of the calibrated NI margin. Full details of this derivation, including the formulas for pooling RD and SE values, are provided in Method S3. SE provides an estimate of the precision of the RD and is used to produce CIs and *P*-values. Abbreviations: M1 = conservative estimate of treatment effect; M2 = acceptable reduction in treatment effect; NI = non-inferiority; RD = risk difference; SE = standard error; STTC = Stroke Thrombolysis Trialists’ Collaboration.

The final reweighted estimate of risk difference for alteplase vs control was 11.70% (95% CI, 6.67–16.73), from which we used the 95–50–95 fixed margin rule^4^ to derive the non-inferiority margin. The conservative estimate (lower 95% bound) of the alteplase vs control treatment effect (M1) was therefore 6.67%, with a 50% reduction of this effect (M2) corresponding to a risk difference of 3.33%. The calibrated non-inferiority margin for comparing alternative interventions to alteplase was therefore set at −3.33% (Figure 5). This calibrated margin broadly agrees with the margin originally chosen by TASTE investigators and the European Stroke Organisation’s clinically recommended margin derived from subject-matter experts (−3% in both cases).^3^^,^^15^

If we had not performed this calibration process and instead used the distribution of patients within the STTC dataset, the estimated risk difference for alteplase vs control would have been 6.76% (95% CI, 4.02–9.50). Following the same 95–50–95 fixed margin rule as above, the conservative estimate for the control to alteplase treatment effect (M1) would have been 4.02%, with a 50% reduction in treatment effect (M2) resulting in a risk difference of 2.01%. The corresponding non-inferiority margin would therefore have been set at −2.01%. This overly stringent non-inferiority margin is a result of the STTC dataset containing a larger number of participants in whom alteplase is less effective, relative to the TASTE trial population.

The primary endpoint results of TASTE were 3% (95% CI, −3.3 to 10), which would have put the results of TASTE (−3.3%) directly on the boundary of the calibrated margin (−3.33%). In anticipation of this shift, the protocol had a pre-specified fixed margin of −3%, compared with a non-calibrated margin of −2%. Therefore, whilst this seems like a small correction (−2% to −3% to −3.33%), it would have been sufficient to put the results from the intention-to-treat population from TASTE directly on the boundary of the margin (−3.3%). From a numerical perspective, this could have led to a positive trial outcome, in the context of a reasonable scenario of comparable non-inferiority.

Appendix S2 is an editable calculator that can be used for the whole procedure of estimating calibrated non-inferiority margins. Step 3 of this file includes a toggle that enables the M2 sensitivity to be amended to a different percentage reduction in treatment effect.

The results of our sensitivity analyses using the TASTE data confirmed the robustness of the proposed 4-step approach, regardless of the number of subgroups, providing that the results are numerically calculable (≥ 1 event in each subgroup for each treatment). The results were performed using SAS version 9.4 and are reported in Table S3.

## Discussion

Our conceptual approach to estimating calibrated non-inferiority margins is a simple and pragmatic way to account for shifts in patient populations. It does not make burdensome statistical assumptions and only requires commonly reported subgroup-specific treatment-effect estimates. These strengths make it a pragmatic alternative to the existing statistical approaches identified by the European Commission for Health Technology Assessments.^8^^,^^20^

The procedure that we developed to support this conceptual approach is readily applied in practice and allows stroke researchers to calibrate historical treatment effects to reflect current patient populations and standards of care, maintaining validity without requiring burdensome statistical assumptions or complete individual patient data. This procedure uses data-dependent weights but is fully pre-specifiable and can be described within a study protocol. Such pre-specification, combined with the fact that it is conducted irrespective of treatment allocation and without the need to examine outcome data, means it has no effect on type-I error. We envisage this procedure being applied in a blinded manner after the last participant has been randomised but prior to the study database lock.

Pre-specifying a procedure for determining a calibrated non-inferiority margin based on a recruited participant population, rather than pre-specifying a non-inferiority margin value ahead of time, may be considered a form of adaptive trial component; as such, pre-specified procedures are consistent with working definitions used to describe adaptive clinical trials.^21^ It is compatible with established adaptive trial designs that use interim analyses for early stopping or sample-size re-estimation, as the procedure may be applied to produce a calibrated non-inferiority margin that matches the study population at the time of interim analysis. Furthermore, if a data safety and monitoring committee is interested in questions of treatment efficacy in studies where population shift is possible, this method could be used to provide the committee with an appropriate non-inferiority margin at each meeting.

Our pre-specified procedure has already been used in the re-analysis of the AcT study,^7^^,^^22^ which subsequently led to approval of tenecteplase 25 mg in the early time window in Europe and many other countries.^7^ Our proposed method outlined in this article was conceptualised as a pragmatic solution to account for substantial differences in the onset-to-treatment time between the AcT and STTC studies. This difference in the distribution was also observed between TASTE and STTC (see Figure 3). Had this pre-specified approach in TASTE been used in an adaptive manner, the main trial results could have been positive. In this example, our approach was shown to derive a calibrated non-inferiority margin that was consistent with the European Stroke Organisation’s clinically recommended margin, a consensus-based estimate from subject-matter experts. Our approach, which is applicable to a variety of clinical contexts, including the development of biosimilars, could be used as a decision support tool for future expert consensus meetings.

A key strength of our conceptual approach is that, unlike existing methods,^8^^,^^20^ it only requires subgroup-specific treatment-effect estimates. While we used individual patient data to streamline the development of the illustrated practical procedure, it would not have been necessary for execution in practice. The required subgroup analyses from historical RCT data may be conducted in collaboration with historical data holders, without the need for individual patient data to be shared. In addition, future non-inferiority trials comparing an experimental treatment vs alteplase may skip this step entirely by reusing the subgroup analyses results reported in this manuscript to create a non-inferiority margin calibrated to their trial population, rather than that of TASTE. This strength resolves the significant practical and statistical limitations that have prevented the use of calibrated non-inferiority margins in stroke research. However, it does not eliminate the need for collaboration between non-inferiority trialists and historical data holders. Such collaboration is fundamental to good research practice, and our proposed approach is intended to streamline this collaboration.

While we demonstrated our method using risk differences as the treatment-effect-size measure, it may be readily applied to other effect measures such as difference in means. The primary statistical assumption made by our method is that the estimate of the treatment effect is normally distributed (ie, the Central Limit Theorem applies), which is true for all commonly used effect-size measures in stroke, even with a moderate sample size. However, care should be taken for non-collapsible effect-size measures such as odds ratios and hazard ratios. Non-collapsible effect-size measures, when estimated on a complete dataset, will produce a different result than what would have been produced had this estimation been performed on subgroups and then averaged.^23^ Therefore, our proposed method may result in a less conservative treatment—and corresponding non-inferiority margin—for such effect-size measures. Whilst collapsibility is a topic of discussion in medical statistics,^24^ the conventional approach for measures such as risk ratios and odds ratios is to convert to the log-scale for all computations.^25^ We demonstrate this in Appendix S2 by repeating our illustrative example using risk ratios as the effect-size measure.

It is important to note that a calibrated non-inferiority margin is only required if the distribution of effect-modifying (“predictive”) covariates has changed. In the context of binary or dichotomised outcomes, any covariate that changes baseline outcome prevalence (“prognostic” covariates) can be considered effect-modifying if more than one representation of the treatment effect (eg, risk difference and risk ratio) is to be considered. If the risk difference is the same in 2 subgroups but one of these subgroups has a lower outcome prevalence, then, by definition, the risk ratio must be different. Likewise, the same risk ratio across 2 subgroups with different baseline prevalences will result in different risk differences. We illustrate this further in Appendix S2. As binary outcomes are common in stroke, any shift in an important covariate justifies the use of a calibrated non-inferiority margin.

One challenge introduced by pre-specifying a procedure for determining a calibrated non-inferiority margin (rather than setting the margin directly) is that power calculations become more difficult. Sample-size estimation for non-inferiority trials typically uses the value of the non-inferiority margin, which, in this case, would not be known until after the trial had started. There are several ways this could be addressed. First, if a good estimation of the distribution of the study population is available beforehand, a preliminary value of the margin could be determined for the purposes of sample-size estimation, with the sample size selected to ensure that statistical power will fall within an acceptable band under a range of plausible scenarios. Alternatively, our approach could be combined with adaptive sample-size re-estimation procedures to ensure appropriate statistical power, based on the observed population characteristics at the time of interim analysis.^21^ Such a combination is a topic for future research.

Another topic for further exploration is to compare in detail this method with other more sophisticated approaches, especially with regard to the bias and precision achieved by each method.

Our conceptual approach offers opportunities for further research beyond developing improved sample-size estimation procedures. Our procedure determined weights irrespective of treatment assignment and was developed in the context of 1:1 treatment allocation ratios. Further research is needed to explore how this approach may be applied in trials that use uneven allocation ratios.

## Conclusion

In summary, it is well recognised that stroke trialists need to account for shifts in patient populations when estimating non-inferiority margins. We proposed a conceptual approach that addresses this need, supported by a fully pre-specifiable procedure implementing the concept. This was demonstrated to derive a calibrated non-inferiority margin for comparing tenecteplase with alteplase that accounts for changes in the patient population.

## Supplementary Material

aakaf022_REVISED2_PopAdjNIMargin_ESJ_Supplemental_Materials_8Nov_clean

aakaf022_REVISED_PopAdjNIMargin_Supplementary_Appendix_4_20Oct

## Data Availability

All data access requests should be made to the data holders of the respective studies included in this analysis (TASTE and STTC).

## Trial registration

Not applicable because this was a statistical analysis of historical data from completed studies.
