# Correction: Calibrated non-inferiority margin: a new pragmatic method to account for population shift in stroke trials

**DOI:** 10.1093/esj/aakag107

**Published:** 2026-09-01

This is a correction to: Nuala Peter, Hannah Johns, Bruce C V Campbell, Bijoy Menon, Mark W Parsons, Leonid Churilov, Calibrated non-inferiority margin: a new pragmatic method to account for population shift in stroke trials, *European Stroke Journal*, Volume 11, Issue 1, January 2026, aakaf022, https://doi.org/10.1093/esj/aakaf022

The graphical repesentation in panel B of Figure 3 of the data that derive from Table 1 was incorrect: the revised version now displays these data correctly.

Figure 3 (panel B) and caption should read:


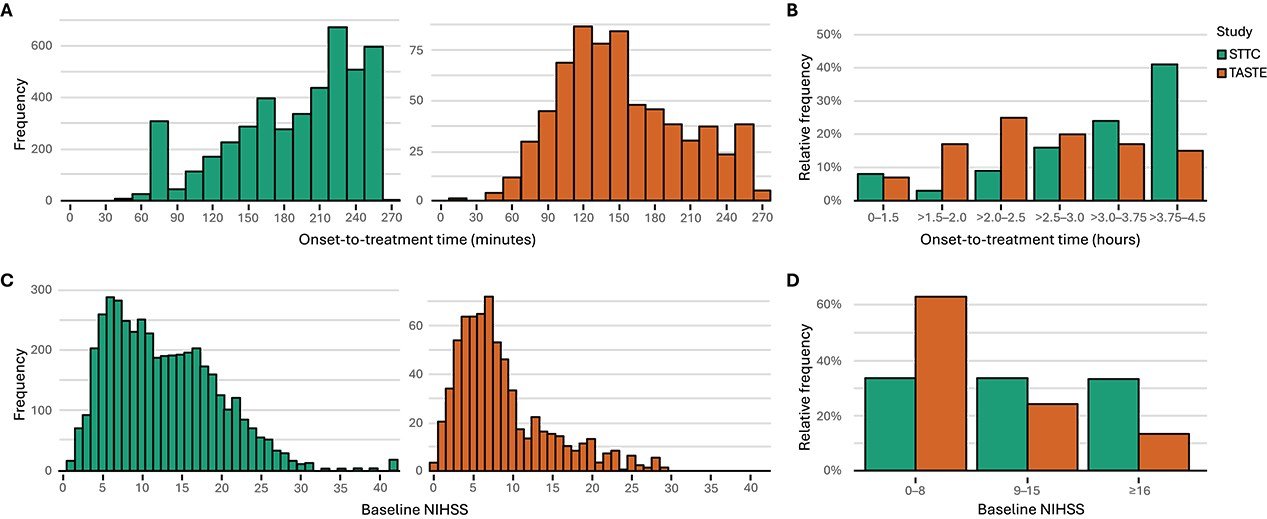


Distribution of prognostic factors in the historical meta-analysis and the new trial. Panels A and B pertain to both STTC and TASTE datasets, showing distribution across OTT categories. Panels C and D pertain to both STTC and TASTE datasets, showing distribution across baseline NIHSS categories. Abbreviation: STTC = Stroke Thrombolysis Trialists’ Collaboration.

instead of:


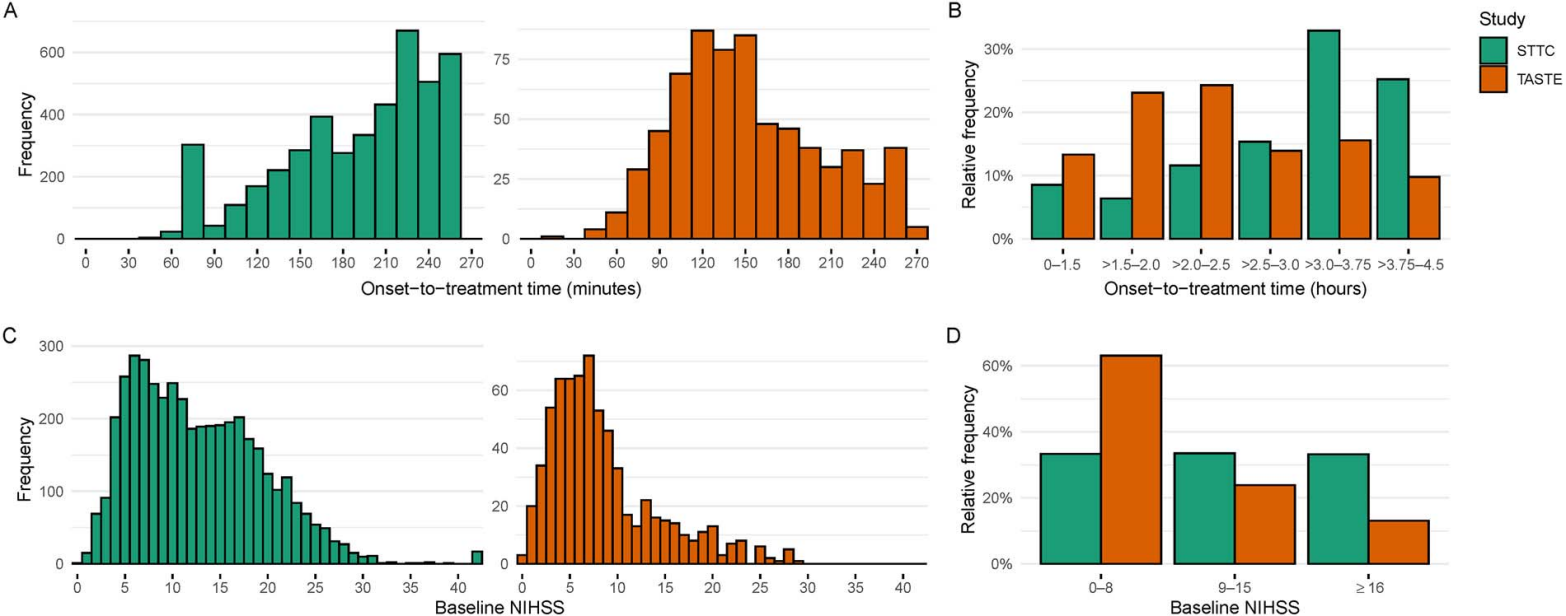


Distribution of prognostic factors in the historical meta-analysis (STTC data; panels A and B) and the new trial (TASTE; panels C and D). Panels A and C present the frequency of patients within categories defined by onset-to-treatment time (15-minute time bins) and baseline NIHSS (single-unit bins), respectively. Panels B and D present the relative frequency of combined categories for each prognostic factor; categories were large enough to estimate subgroup-specific treatment effects within the STTC dataset. Abbreviation: STTC = Stroke Thrombolysis Trialists’ Collaboration.

The emendations have been made to the paper.

