# Supplementary material for: Calibrated non-inferiority margin: a new pragmatic method to account for population shift in stroke trials
Source: Eur Stroke J. 2026 Jan 1;11(1):aakaf022. doi: 10.1093/esj/aakaf022 (PMC12866653; doi:10.1093/esj/aakaf022)
Supplement: aakaf022_REVISED2_PopAdjNIMargin_ESJ_Supplemental_Materials_8Nov_clean [file aakaf022_revised2_popadjnimargin_esj_supplemental_materials_8nov_clean.docx]

# Supplemental Material

# Calibrated non-inferiority margin: a new pragmatic method to account for population shift in stroke trials

Nuala Peter MSc,^1^ Hannah T. Johns PhD,^2,3^ Bruce C.V. Campbell MD,^4^ Bijoy K. Menon MD,^5^ Mark W. Parsons PhD,^6^ Leonid Churilov PhD^2,3^

^1^Boehringer Ingelheim Pharma GmbH & Co. KG, Biberach an der Riss, Germany

^2^Melbourne Medical School, University of Melbourne, Melbourne, Victoria, Australia

^3^Australian Stroke Alliance, Melbourne Brain Centre, Royal Melbourne Hospital, Parkville, Victoria, Australia

^4^Department of Medicine and Neurology, Melbourne Brain Centre, Royal Melbourne Hospital, University of Melbourne, Parkville, Victoria, Australia

^5^Calgary Stroke Program, University of Calgary Cumming School of Medicine, Calgary, Alberta, Canada

^6^Department of Neurology, Liverpool Hospital, Liverpool, New South Wales, Australia

# Supplemental Appendix 1: Background to non-inferiority trials

An active comparator is a clinical intervention that has been previously demonstrated to be superior to control. Non-inferiority trials claim success if the difference in outcomes between an experimental treatment and the active comparator is significantly above a pre-specified non-inferiority margin. The non-inferiority margin quantifies the maximum acceptable worsening of outcome for the experimental treatment when compared with an active comparator.

The intricate nature of conducting and interpreting non-inferiority trials has led to recently published reviews within stroke literature on the topic.^1,2^ Saver and Mistry^2^ identified three types of non-inferiority trials, each with different purposes and different interpretations for their non-inferiority margin: indistinguishability, reasonable comparability, and balanced advantages.

The methods used for deriving the non-inferiority margin vary according to the type of non-inferiority trial. For trials seeking to claim indistinguishability (i.e. where an acceptable reduction in efficacy of the experimental treatment compared with an active comparator is small enough to not be clinically relevant), the non-inferiority margin is set based on the minimal clinically important difference for the chosen outcome, which could also be the same as the expert consensus. For the other two cases, the margin may be estimated based on expert consensus,^3^ and/or using historical data and the commonly applied 95–95 fixed margin approach,^4^ where a certain percentage (e.g. 50%) reduction in treatment effect demonstrated by the active comparator compared with control is considered acceptable. A reduction of more or less than 50% may be considered acceptable in different settings depending on a range of factors, including adverse events, ease of administration, and cost.^4^

Irrespective of which of the three types of non-inferiority is being considered, the non-inferiority margin relies on the constancy assumption, i.e. that the treatment effect of an active comparator to control in the non-inferiority trial population has not changed from the historical context used to derive the margin.^1^ A detailed review of methodologic assumptions for non-inferiority trials is beyond the scope of this paper due to its nature and is provided in literature.^5,6^ Cavalcante and colleagues^1^ recently highlighted that the constancy assumption is likely to be violated in stroke, where considerable changes in standards of care and patient demographics have been observed. While emphasized in the context of non-inferiority margins derived from historical data, this consideration equally applies to non-inferiority margins derived from expert consensus. Provided that the treatment effect of an active comparator varies across patient subgroups, any non-inferiority margin derived by expert consensus in the process of designing a non-inferiority trial implicitly makes assumptions about the relative frequency of these subgroups in the participant sample for the non-inferiority trial being designed. Any trial that interprets the non-inferiority margin as an acceptable reduction in treatment effect of the active comparator compared with control relies on the constancy assumption; if such a margin is determined without accounting for changes in patient subpopulations, the results of any analysis using it may be biased and misleading.^7^ This challenge is exacerbated by the requirement to pre-specify the non-inferiority margin during trial design purely based on the assumed participant sample, as it is not possible to exactly know the resulting distribution of effect-modifying covariates in advance at the design stage. Nevertheless, a good estimate could be anticipated during the design phase.

# Supplemental Methods 1: Complete and unambiguous description of procedure for deriving calibrated non-inferiority margins

For the sake of brevity, in the remainder of this supplement, we refer to the study that compared control to active comparator as the “historical study”, and the study comparing an experimental treatment to an active comparator as the “non-inferiority study”.

The initial step for developing a calibrated non-inferiority margin is to identify effect modifiers with a shift in distribution. This may be performed by comparing the distribution of important covariates across the historical study to the non-inferiority study.

**Step 1: Determine subgroups**

To create subgroups, first take all selected covariates from the initial step and create clinically meaningful bins. The subgroups used in this procedure are based on the full-factorial combination of each clinical variable. We note the total number of subgroups developed in this way as $K$.

Each subgroup should be sufficiently large within the historical study to satisfy the Central Limit Theorem, the underlying statistical mechanism that allows for the normal distribution to be used to estimate confidence intervals and standard errors. A common guiding principle is that each arm within each subgroup should contain ≥15 observations and ≥1 event.

**Step 2: Perform subgroup analyses**

For each subgroup $i$, estimate the treatment effect size $\delta_{i}$ and corresponding standard error $\sigma_{i}$ using data from the historical study. If the treatment effect is a ratio-based scale such as a risk ratio, hazard ratio, or odds ratio, $\delta_{i}$ refers to the log-scale treatment effect.

The estimated treatment effects may be adjusted for other important prognostic covariates or unadjusted, provided that this analysis choice is consistent with the analyses that will be performed in the non-inferiority study.

**Step 3: Determine weights**

The weight $w_{i}$ for subgroup $i$ is given by the proportion of data within the non-inferiority study, that is

$$w_{i}=n_{i}/N$$

Where $n_{i}$ is the number of participants from the non-inferiority study in subgroup $i$ and $N$ is the total sample size in the non-inferiority study, i.e. $N=n_{1}+n_{2}+\cdots+n_{K}$. Note that the sum of all weights $w_{i}$ must equal 1.

**Step 4: Pool the weighted estimators and calculate calibrated non-inferiority margin**

The pooled treatment effect $\bar{\delta}$ and corresponding standard error $\bar{\sigma}$ are calculated by

$$\bar{\delta}= \sum_{i=1}^{K} w_{i}\delta_{i}=w_{1}\delta_{1}+ w_{2}\delta_{2}+\ldots+ w_{K}\delta_{K}$$

$$\bar{\sigma}=\sqrt{\sum_{i=1}^{K} \left( w_{i}\sigma_{i} \right)^{2}}=\sqrt{\left( w_{1}\sigma_{1} \right)^{2}+\left( w_{2}\sigma_{2} \right)^{2}+\ldots+\left( w_{K}\sigma_{K} \right)^{2}}$$

The 95% confidence interval for the pooled treatment effect is then given by

$$\bar{\delta}\pm Z_{1-\frac{0.05}{2}}\times\bar{\sigma}$$

Where $Z_{1-\frac{0.05}{2}}\approx1.959964$ is the critical value for the normal distribution used for estimating 95% confidence intervals.

Assuming that this pooled treatment effect is significant, the conservative estimate of the treatment effect $M1$ is given by whichever end of the 95% confidence interval is closest to the null value (on a log scale, the null value for, e.g. hazard ratios, risk ratios, and odds ratios, is 0 as $\text{log}\left( 1 \right)=0$).

For example:

$$M1=\text{sgn}\left( \bar{\delta} \right)\times\text{min}\left\{ \begin{matrix} \left| \bar{\delta}-Z_{1-\frac{0.05}{2}}\times\bar{\sigma} \right| & \begin{matrix} \\ \\ , \end{matrix} & \left| \bar{\delta}+Z_{1-\frac{0.05}{2}}\times\bar{\sigma} \right| \end{matrix} \right\}$$

If this treatment effect is allowed to be reduced by $r$%, then the minimum acceptable treatment effect $M2$ is given by

$$M2=\frac{(100-r)}{100}\times M1$$

And the non-inferiority margin for comparing an experimental treatment to the active comparator is given by

$$\text{NIM}=-M2$$

If the treatment effect is allowed to be reduced by 50% following the 95–50–95 rule, then this is simplified, and the non-inferiority margin is given by $\text{NIM}=-0.5\times M1$

If the treatment effect was considered on a log scale, the non-inferiority margin may be exponentiated at this point to provide the non-inferiority margin on a natural scale.

# Supplemental Methods 2: Derivation of risk ratio non-inferiority margin using the STTC dataset

**Step 1: Determine subgroups**

This step is identical to the step performed in the manuscript.

**Step 2: Perform subgroup analyses**

We estimated the risk ratio and corresponding standard error for each subgroup, given below.

|  |  | **mRS 0–1** | |  |
| --- | --- | --- | --- | --- |
| OTT time (hours) | Baseline NIHSS | Alteplase | Control | RR (95% CI) |
|  | 0–8 | 28/38 (73.68%) | 17/27 (62.96%) | 1.170 (0.828–1.654) |
| 0–1.5 | 9–15 | 27/55 (49.09%) | 17/57 (29.82%) | 1.646 (1.018–2.662) |
|  | 16–42 | 14/78 (17.95%) | 10/75 (13.33%) | 1.346 (0.638–2.841) |
|  | 0–8 | 12/23 (52.17%) | 3/18 (16.67%) | 3.130 (1.037–9.448) |
| >1.5–2 | 9–15 | 6/16 (37.50%) | 4/25 (16.00%) | 2.344 (0.781–7.031) |
|  | 16–42 | 7/34 (20.59%) | 1/35 (2.86%) | 7.206 (0.936–55.498) |
|  | 0–8 | 25/46 (54.35%) | 23/51 (45.10%) | 1.205 (0.806–1.802) |
| >2–2.5 | 9–15 | 19/76 (25.00%) | 11/55 (20.00%) | 1.250 (0.648–2.410) |
|  | 16–42 | 8/79 (10.13%) | 3/83 (3.61%) | 2.802 (0.771–10.184) |
|  | 0–8 | 71/112 (63.39%) | 47/97 (48.45%) | 1.308 (1.020–1.678) |
| >2.5–3.0 | 9–15 | 28/112 (25.00%) | 28/110 (25.45%) | 0.982 (0.624–1.545) |
|  | 16–42 | 14/118 (11.86%) | 12/129 (9.30%) | 1.275 (0.615–2.645) |
|  | 0–8 | 104/175 (59.43%) | 93/178 (52.25%) | 1.137 (0.944–1.370) |
| >3–3.75 | 9–15 | 44/164 (26.83%) | 41/166 (24.70%) | 1.086 (0.753–1.567) |
|  | 16–42 | 14/168 (8.33%) | 12/191 (6.28%) | 1.326 (0.631–2.787) |
|  | 0–8 | 218/349 (62.46%) | 178/338 (52.66%) | 1.186 (1.042–1.350) |
| >3.75–4.5 | 9–15 | 88/304 (28.95%) | 97/321 (30.22%) | 0.958 (0.752–1.220) |
|  | 16–42 | 17/215 (7.91%) | 11/243 (4.53%) | 1.747 (0.837–3.646) |

CI, confidence internal; mRS, modified Rankin Scale; NIHSS, National Institutes of Health Stroke Scale; OTT, onset-to-treatment; RR, risk ratio.

**Step 3: Determine weights**

This step is identical to the step performed in the manuscript.

**Step 4: Pool the weighted estimators and calculate calibrated non-inferiority margin**

Following the procedure for pooling weights outlined above, the calibrated effect of alteplase when compared to control was a risk ratio of 1.465 (95% confidence interval: 1.238–1.732). The conservative margin for treatment effect is therefore $M1=1.238$. If a 50% reduction in treatment effect is allowed, the minimum preserved treatment effect is $M2=\exp\left( 0.5\times\ln\left( 1.238 \right) \right)=1.113$. The non-inferiority margin is therefore given by $\text{NIM}=\exp\left( -0.5\times\ln\left( 1.238 \right) \right)=0.899$.

# Supplemental Methods 3: Demonstration that all covariates are “effect-modifying” on at least one scale for binary outcomes

An effect-modifying covariate is defined as one where the presence or absence of the covariate changes the magnitude of the effect of an intervention. Consider a simple case where an intervention has a risk difference of 0.2, and a binary covariate that does not change the treatment effect of this intervention increases the probability of having a good outcome by 0.1 if the covariate is present. The following table summarizes this information.

| **Binary covariate** | **Probability of good outcome if given control** | **Probability of good outcome if given intervention** | **Risk difference** |
| --- | --- | --- | --- |
| Absent | 0.1 | 0.3 | 0.2 |
| Present | 0.2 | 0.4 | 0.2 |

By definition, this covariate is not effect-modifying, as the risk difference is unchanged by its presence or absence. If, instead of estimating the risk difference, we estimated the risk ratio or odds ratio, we would see that the effect size is changed by the presence of the covariate, even though the probability of good outcome is identical in all scenarios.

| **Binary covariate** | **Probability of good outcome if given control** | **Probability of good outcome if given intervention** | **Risk difference** | **Risk ratio** | **Odds ratio** |
| --- | --- | --- | --- | --- | --- |
| Absent | 0.1 | 0.3 | 0.2 | 3.0 | 3.857 |
| Present | 0.2 | 0.4 | 0.2 | 2.0 | 2.667 |

Similar arguments may be made by beginning with a scenario where a covariate is not effect-modifying in terms of the risk ratio or is not effect-modifying in terms of the odds ratio, and demonstrating that it is effect-modifying in terms of the other two effect-size measures.

# Supplemental Table 1: Procedure for deriving calibrated non-inferiority margin for TASTE, using historical STTC data

| **Step** | **Details** |
| --- | --- |
| Initial | We identified onset-to-treatment time, age, and baseline NIHSS as important prognostic factors that could bias the estimation of the non-inferiority margin in the presence of systematic differences between the STTC and TASTE study populations, and selected any of them where such systemic differences were observed. |
| Step 1 | We created granular bins for the above variables selected for calibration, and, in consultation with clinical experts, merged these bins into clinically interpretable groups. We required each group to contain an adequate sample size to facilitate treatment-effect estimates that do not make statistical assumptions in the STTC study population to reliably estimate treatment control to active comparator treatment effects. This process was performed separately for each selected variable.  The final subgroups were formed from the full-factorial combination of bins for all selected variables, selected such that the total number of subgroups was sufficiently small to provide reasonable subgroup-specific treatment-effect estimates in the STTC dataset. |
| Step 2 | We estimated the control to active comparator treatment effect within each of these subgroups in the STTC dataset using risk differences. |
| Step 3 | We calculated subgroup weights using the number of participants in each subgroup within the TASTE dataset. |
| Step 4 | We estimated the calibrated reweighted treatment effect (risk difference) of alteplase vs control for the TASTE dataset by applying our proposed reweighting procedure. We derived the calibrated non-inferiority margin based on this risk difference using the 95–50–95 fixed margin rule, allowing for a maximum 50% reduction in the calibrated efficacy of alteplase compared with control. The 95–50–95 fixed margin rule uses the lower tail of the 95% confidence interval (M1 using FDA terminology^7^ and in Figure 1) as a conservative estimate of the control to active comparator treatment effect and then allows for a 50% reduction of this effect (M2 using FDA terminology^7^ and in Figure 1). Non-inferiority is claimed if the lower tail of the 95% confidence interval for the experimental treatment to active comparator treatment effect is above this reduced treatment effect. |

FDA, U.S. Food and Drug Administration; NIHSS, National Institutes of Health Stroke Scale; STTC, Stroke Thrombolysis Trialists’ Collaboration.

# Supplemental Table 2: Distribution of weights determined from TASTE for each of the subgroups defined in Step 1

|  | **Baseline NIHSS** | | | | | | | | | | | |
| --- | --- | --- | --- | --- | --- | --- | --- | --- | --- | --- | --- | --- |
| **Onset-to-treatment time (hours)** | **0–8** | | | **9–15** | | | **≥16** | | | **Total** | | |
|  | TASTE |  | STTC | TASTE |  | STTC | TASTE |  | STTC | TASTE |  | STTC |
| 0–1.5 | 0.044 |  | 0.015 | 0.015 |  | 0.026 | 0.007 | < | 0.035 | **0.066** |  | **0.076** |
| >1.5–2.0 | 0.104 | > | 0.009 | 0.049 | > | 0.009 | 0.016 |  | 0.016 | **0.169** | **>** | **0.034** |
| >2.0–2.5 | 0.147 | > | 0.022 | 0.061 |  | 0.03 | 0.039 |  | 0.037 | **0.247** |  | **0.089** |
| >2.5–3.0 | 0.135 |  | 0.048 | 0.028 |  | 0.051 | 0.034 |  | 0.057 | **0.197** |  | **0.156** |
| >3.0–3.75 | 0.104 |  | 0.081 | 0.049 |  | 0.076 | 0.016 | < | 0.082 | **0.169** |  | **0.239** |
| >3.75–4.5 | 0.099 |  | 0.158 | 0.034 | < | 0.143 | 0.019 | < | 0.105 | **0.152** |  | **0.406** |
| **Total** | **0.633** |  | **0.333** | **0.236** |  | **0.335** | **0.131** |  | **0.332** | **1** |  | **1** |

> indicates subgroup at least three times more frequent in the TASTE dataset.
< indicates subgroup at least three times more frequent in the STTC dataset.

NIHSS, National Institutes of Health Stroke Scale; STTC, Stroke Thrombolysis Trialists’ Collaboration.

# Supplemental Table 3: Systematic sensitivity analyses using propensity score methods and alternative grouping schemes

| **Method** ^8, 9, 10^ | **RD (%) [95% CI]** | **M1 / M2 / NIM**  **with 50% preservation, (%)** |
| --- | --- | --- |
| **PSS with equally sized subgroups of various strata size** | |  |
| 2 equally sized subgroups | 11.05 [6.63–15.48] | 6.63 / 3.32 / –3.32 |
| 3 equally sized subgroups | 10.01 [5.32–14.70] | 5.32 / 2.66 / –2.66 |
| 4 equally sized subgroups | 11.76 [7.03–16.48] | 7.03 / 3.52 / –3.52 |
| 5 equally sized subgroups | 11.65 [6.91–16.40] | 6.91 / 3.46 / –3.46 |
| 6 equally sized subgroups | 10.74 [5.93–15.54] | 5.93 / 2.97 / –2.97 |
| 7 equally sized subgroups | 11.62 [6.79–16.45] | 6.79 / 3.40 / –3.40 |
| 8 equally sized subgroups | 11.47 [6.65–16.30] | 6.65 / 3.33 / –3.33 |
| 9 equally sized subgroups | 12.08 [7.16–16.99] | 7.16 / 3.58 / –3.58 |
| 10 equally sized subgroups | 11.63 [6.73–16.54] | 6.73 / 3.37 / –3.37 |
| 11 equally sized subgroups | 11.48 [6.63–16.33] | 6.63 / 3.32 / –3.32 |
| 12 equally sized subgroups | 10.78 [5.94–15.63] | 5.94 / 2.97 / –2.97 |
| 13 equally sized subgroups | 11.12 [6.17–16.06] | 6.17 / 3.09 / –3.09 |
| 14 equally sized subgroups | 11.55 [6.66–16.44] | 6.66 / 3.33 / –3.33 |
| 15 equally sized subgroups | 10.42 [5.45–15.40] | 5.45 / 2.73 / –2.73 |
| 16 equally sized subgroups | 10.28 [5.26–15.30] | 5.26 / 2.63 / –2.63 |
| 17 equally sized subgroups | 11.15 [6.23–16.07] | 6.23 / 3.12 / –3.12 |
| 18 equally sized subgroups | 11.00 [6.14–15.86] | 6.14 / 3.07 / –3.07 |
| 19 equally sized subgroups | 10.38 [5.57–15.20] | 5.57 / 2.79 / –2.79 |
| 20 equally sized subgroups | 10.93 [6.07–15.78] | 6.07 / 3.04 / –3.04 |
| 21 equally sized subgroups | 10.82 [6.05–15.59] | 6.05 / 3.03 / –3.03 |
| 22 equally sized subgroups | 10.41 [5.60–15.22] | 5.60 / 2.80 / –2.80 |
| **Propensity score ATT weights** |  |  |
| Without trimming | 10.92 [3.44–18.40] | 3.44 / 1.72 / –1.72 |
| With trimming (limited to 1–99 percentiles) | 10.92 [3.44–18.40] | 3.44 / 1.72 / –1.72 |
| With trimming (limited to 2.5–97.5 percentiles) | 10.93 [3.40–18.47] | 3.40 / 1.70 / –1.70 |
| With trimming (limited to 5–95 percentiles) | 11.29 [3.55–19.03] | 3.55 / 1.78 / –1.78 |
| With trimming (limited to 10–90 percentiles) | 12.08 [3.65–20.52] | 3.65 / 1.83 / –1.83 |
| With trimming (limited to 15–85 percentiles) | 12.21 [2.88–21.53] | 2.88 / 1.44 / –1.44 |
| With trimming (limited to 20–80 percentiles) | 14.58 [4.24–24.93] | 4.24 / 2.12 / –2.12 |
| With trimming (limited to 25–75 percentiles) | 16.36 [4.85–27.87] | 4.85 / 2.43 / –2.43 |
| **4-step approach with various subgroup sizes** | |  |
| 6 subgroups  OTT time, hours (3 levels): 0–1.5; >1.5–2; >2  Baseline NIHSS (2 levels): 0–8; ≥9 | 10.97 [6.87–15.07] | 6.87 / 3.44 / –3.44 |
| 9 subgroups  OTT time, hours (3 levels): 0–1.5; >1.5–2; >2  Baseline NIHSS (3 levels): 0–8; 9–15; ≥16 | 11.01 [6.76–15.25] | 6.76 / 3.38 / –3.38 |
| 12 subgroups  OTT time, hours (4 levels): 0–1.5; >1.5–2; >2–3; >3  Baseline NIHSS (3 levels): 0–8; 9–15; ≥16 | 11.93 [7.14–16.72] | 7.14 / 3.57 / –3.57 |
| 18 subgroups  OTT time, hours (5 levels): 0–1.5; >1.5–2; >2–2.5; 3–3.75; 3.75–4.5  Baseline NIHSS (3 levels): 0–8; 9–15; ≥16 | 11.70 [6.67–16.73] | 6.67 / 3.33 / –3.33 |

CI, confidence interval; PSS, propensity score stratification; RD, risk difference: NIM, non-inferiority margin; ATT, among-the-treated; PSW, propensity score weighting; NIHSS, National Institutes of Health Stroke Scale; OTT, onset-to-treatment.

***References***

1. Cavalcante F, Treurniet KM, Kappelhof M, et al. Understanding noninferiority trials: what stroke specialists should know. *Stroke* 2025; 56: 543-552.

2. Saver JL and Mistry E. Time to recognize three classes of non-inferiority trial margins. *J Neurointerv Surg* 2023; 15: 2-4.

3. Alamowitch S, Turc G, Palaiodimou L, et al. European Stroke Organisation (ESO) expedited recommendation on tenecteplase for acute ischaemic stroke. *Eur Stroke J* 2023; 8: 8-54.

4. Schumi J and Wittes JT. Through the looking glass: understanding non-inferiority. *Trials* 2011; 12: 106.

5. Guo JD, Gehchan A and Hartzema A. Selection of indirect treatment comparisons for health technology assessments: a practical guide for health economics and outcomes research scientists and clinicians. *BMJ Open* 2025; 15: e091961.

6. Peter N, Danays T, Pagès A, et al. Intravenous tenecteplase compared with alteplase for acute ischemic stroke in Canada (AcT): bridging the gap from academia to regulatory approval. *Stroke Vasc Interv Neurol* 2025; 5: e001705.

7. U.S. Food and Drug Administration. Guidance for industry: Non-inferiority clinical trials to establish effectiveness, <https://www.fda.gov/media/78504/download> (2016, accessed 5 August 2024)

8. Zhang Z, Nie L, Soon G and Hu Z. New methods for treatment effect calibration, with applications to non-inferiority trials. *Biometrics* 2016; 72: 20-29. 20150913. DOI: 10.1111/biom.12388.

9. Nie L, Zhang Z, Rubin D and Chu J. Likelihood reweighting methods to reduce potential bias in noninferiority trials which rely on historical data to make inference. *Ann Appl Stat* 2013; 7: 1796-1813. DOI: 10.1214/13-AOAS655.

10. Rosenbaum PR and Rubin DB. The central role of the propensity score in observational studies for causal effects. *Biometrika* 1983; 70: 41-55. DOI: 10.1093/biomet/70.1.41.
